# Extramammary Paget disease: five perianal case report and treatment options

**DOI:** 10.1093/jscr/rjab262

**Published:** 2021-06-17

**Authors:** Heng Deng, Xiaoli Fang, Ming Li

**Affiliations:** Anorectal Surgery Center, Second Hospital Affiliated Anhui University of Chinese Medicine, Hefei, China; Anorectal Surgery Center, First Hospital Affiliated Anhui University of Chinese Medicine, Hefei, China; Anorectal Surgery Center, First Hospital Affiliated Anhui University of Chinese Medicine, Hefei, China

## Abstract

We present five cases of perianal Paget disease (PPD). Two cases underwent a wide local excision (WLE) of PPD plus skin flap transfer surgery with good curative effect. One case of PPD complicated with mucinous adenocarcinoma underwent a laparoscopic abdominoperineal resection (Miles), which may be the extremes of clinical treatment of the disease. The remaining two cases passed away without surgery after they refused further treatments. This article aims to draw attention to relationship among the correct diagnosis, protection of anal function and treatment options of PPD.

## INTRODUCTION

Extramammary Paget disease (EMPD) is a rare tumor [1]. PPD is one of the rarer subset of EMPD [2, 3], and classified into two categories according to its source, respectively derives from the perianal intraepidermal stem cells and potential malignancies outside the perianal region [4]. The most frequent manifestations are similar to chronic perianal eczema, which can lead to misdiagnosis and worsen the prognosis of patients [5].

Histopathology is the gold standard to confirm correct diagnosis of PPD with the findings of Paget cells containing pale clear cytoplasm, large circles of hyperchromatic nuclei and clusters in the tissue [5].

## CASE REPORT

### Case 1

**Figure 1:**
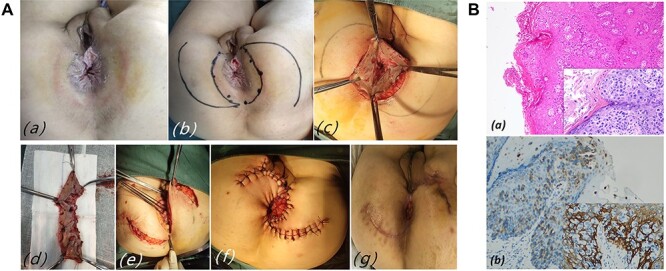
(**A**) Case 1: (**a**) oval erythematous perianal skin lesion with pale secretions, brownish pigmentation and surgical wound; (**b**) scout line determined by negative macroscopic margins > 1 cm (black); (**c**) wide excision with sphincter preservation; (**d**) surgical specimen; (**e**) skin flap transfer surgery; (**f**) Type S sutures the skin wound between the flaps after completion of surgery; (**g**) the incision was basically healed and the mucosa was slightly everted on postoperative Day 60. (**B**) HE stain and IHC in case 1. (**a**) Low and Higher power view show clusters of Paget cells with pleomorphic nuclei and pale cytoplasm in the epidermis (Inset HE×200). (**b**) Paget cells showing strong immunoreactivity for CK7.

A 61 years old female underwent Milligant-Morgant hemorrhoidectomy due to anal itching and stinging sensation, which did not disappear after applying budesonide cream for 3 months. Postoperative pathology coincidentally found Paget cells (Fig. 1B). A 3 × 4 cm oval erythematous perianal skin lesion with pale secretions and surgical wound was noted (Fig. 1A).

A chest and abdominoperineal CT revealed no metastasis or coexisting rectal malignancy. Subsquently, the patient underwent a WLE plus skin flap transfer under combined lumbar and epidural anesthesia. We removed the macroscopic skin lesions, 1 cm of normal skin outside the skin lesions and anal canal skin up to the dentate line. Intraoperative frozen section analysis suggested wide disease extent of 6.1 cm × 6.1 cm and tumor-free margins. Bilateral skin flap transfer surgery was performed for the defect and anal reconstruction. Then, circular anastomosis between the lower end of the rectum and the edge of skin flap was performed without anastomotic stenosis, and type S sutures the skin wound between the flaps to reduce tension. Cefuroxime was given intravenously, fasted for 7 days, and discharged 14 days after the surgery. 1% povidone was used for dressing change once daily. There has been no evidence of recurrence for 2 years.

### Case 2

**Figure 2:**
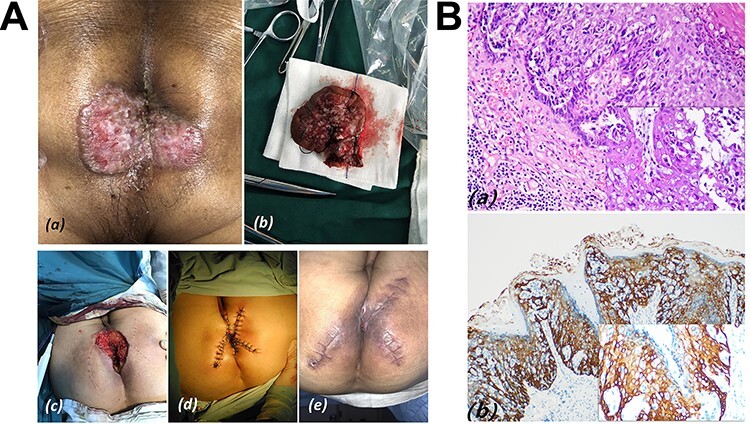
(**A**) Case 2: (**a**) well-defined pink butterfly-like patches interspersed with white secretions above the surrounding skin, and its size was 4 × 5 cm; (**b**) surgical specimen; (**c**) mapping of the incision for skin flap; (**d**) after completion of the wide local excision of PPD plus skin flap transfer surgery; (**e**) the incision was basically healed, nevertheless the reconstructed anal is relatively narrow on postoperative Day 60. (**B**) HE stain and IHC in Case 2; (**a**) clusters of cells with pleomorphic nuclei and pale cytoplasm in the epidermis (inset HE × 200); (**b**) Paget cells showing strong immunoreactivity for CK5/6.

An 81 years old male complained of moist and itchy perianal skin. Well-defined pink butterfly-like patches interspersed with white secretions in the perianal area were found with a size of 4 × 5 cm (Fig. 2A). PPD was diagnosed with to use of phosphorus epithelial cell carcinoma antigen (Fig. 2B).

WLE was performed with negative macroscopic margins of 2 cm to ensure no residual tumor. Mapping the incision of the skin flap was performed, and each flap like a triangle and radially distributed around the defect, which was 1-2 cm thick. Full-thickness suture among the flaps for anal reconstruction were performed. The pathology reported an affected area of 3.5 cm × 4.3 cm and negative resection margins. On postoperative Day 60, the reconstructed anus was found to be relatively stenosed, therefore digital dilatation of the anus was performed once a day until defecation was more easily possible. There has been no evidence of recurrence for 2 years.

### Case 3

**Figure 3:**
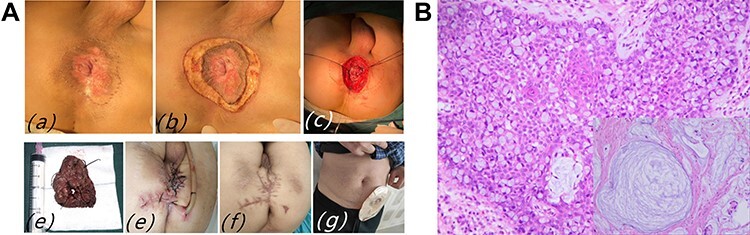
(**A**) Case 3: (**a** and **b**) skin induration around the anus, and scout line determined by negative macroscopic margins > 1 cm; (**c**) wide excision with sphincter preservation; (**d**) surgical specimen; (**e**) after completion of Miles surgery; (**f** and **g**) the incision was basically healed on postoperative Day 60. (**B**) Pathological findings of PPD and mucinous adenocarcinoma: mucinous adenocarcinoma cells with a lot of mucus and pleomorphic nuclei in the center of picture, Paget cell with pleomorphic nuclei and pale cytoplasm.

A 68 years old man was aware of perianal skin induration without pain for 3 years. A reddish oval skin lesion 4 cm in diameter was removed with WLE of PPD plus skin flap transfer (Fig. 3A). PPD was diagnosed with a biopsy, although the margins of the perianal skin incisions at 3, 6, 9 and 12 points at lithotomy were negative in the intraoperative frozen specimens. However postoperative pathological diagnosis found mucinous adenocarcinoma cells in the center of specimens and the anal canal incision edge, furthermore, Paget cells were found in the epidermis of perianal skin (Fig. 3B). Miles surgery was performed under general anesthesia. Negative margins were achieved and no tumor recurrence or metastasis was found after one and a half years of follow-up.

### Case 4

**Figure 4:**
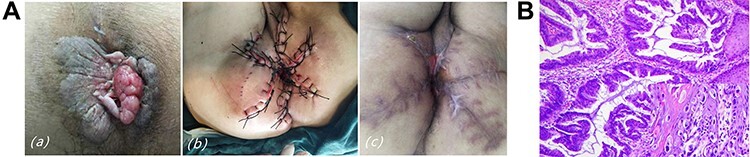
(**A**) Case 4: (**a**) clinical findings: a bleeding lump prolapsed out of anus, abnormal skin lesions around it; (**b**) after completion of the wide local excision of PPD plus skin flap transfer surgery; (**c**) the incision was basically healed on postoperative Day 60. (**B**) Pathological findings of PPD and tubular adenocarcinoma: Paget cell with pleomorphic nuclei and pale cytoplasm, and tubular adenocarcinoma with branched conduits.

A 78 years old man had a 6 months history of painless and bleeding lump prolapsing on defecation, along with pruritus of some abnormal skin lesions (Fig. 4A). Like Case 1, the patient underwent a hemorrhoidectomy. However, two kinds of tumor cells were found like Case 3, tubular adenocarcinoma of rectum and PPD (Fig. 4B). The patient refused Miles operation and accepted WLE of PPD plus skin flap transfer. The incision of the flap transfer was basically healed at 2 months after the second operation. Unfortunately, the patient died of recurrent tubular adenocarcinoma with metastasis 1 year after the surgery.

### Case 5

**Figure 5:**
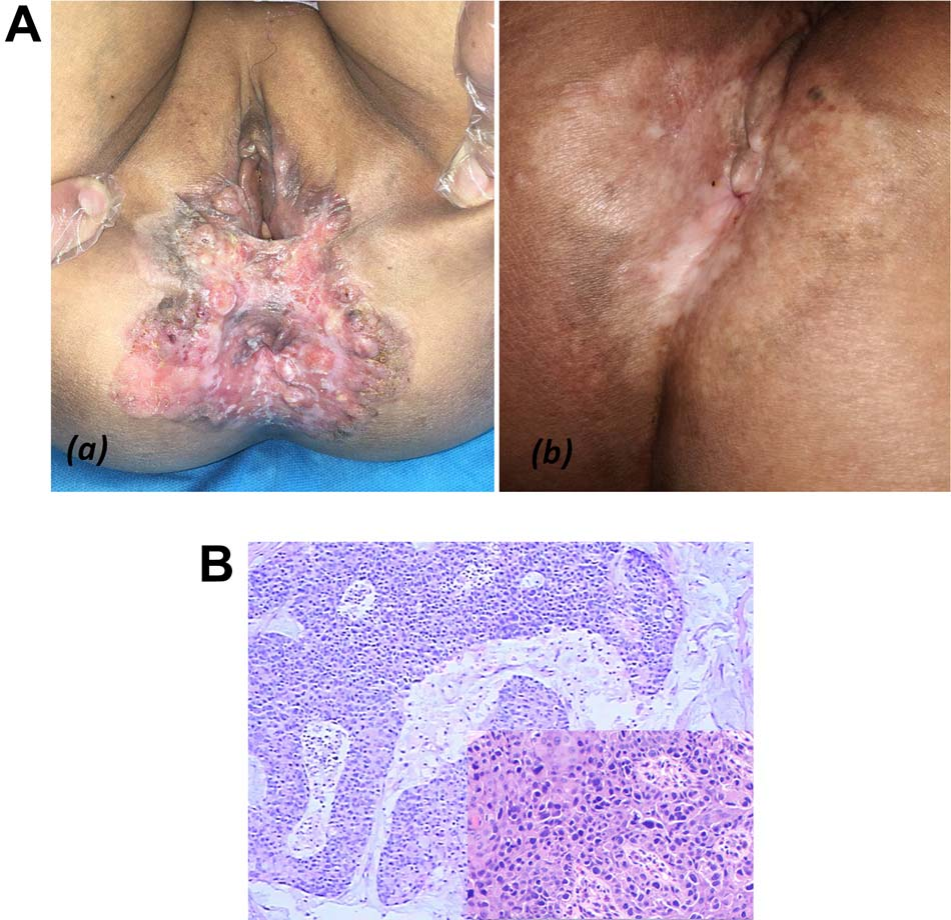
(**A**) Case 5: (**a**) examination of perineal regions with well-defined irregular infiltrating erythema easy to notice; (**b**) perianal local skin lesions were improved after 30 radiotherapy sessions. (**B**) Pathological findings of PPD in left inguinal lymph node: Paget cell with pleomorphic nuclei and pale cytoplasm.

A 67 years old woman with perianal mass for 8 years and perianal skin ulcer with pain for 6 months came to our hospital. Condyloma acuminata was excluded previously, and Paget disease was diagnosed by biopsy. The well-defined erythema surrounding the anus was ~7 × 8 cm in size with surface erosion, scab and impaired the last third of the labia majora (Fig. 5A). Serum tumor markers CEA and CA-199 were elevated. CT found bilateral inguinal lymph node enlargement. Positron emission tomography with computed tomography showed abnormal focal hypermetabolism of perianal skin and bilateral inguinal lymph nodes. A biopsy of left inguinal lymph node revealed metastatic adenocarcinoma (Fig. 5B). The patient refused surgery and decided to seek radiotherapy. After 30 radiotherapy sessions lasting 6 months and a total radiation dose 60 Gy, perianal local symptoms and skin lesions improved significantly. However the patient died of metastasis a year later.

## DISCUSSION

WLE of PPD is a feasible option [6]. But, the circumferential skin defect leads to anal stricture and esthetic dissatisfaction [7]. Therefore, WLE plus skin flap transfer may be a better choice [8]. In our five cases followed up by telephone, three patients accepted WLE plus skin flap transfer without recurrence, and two patients eventually died after they did not completely accept our treatment.

Paget cells can metastasize horizontally or vertically [9]. Negative macroscopic margins > 1 cm with sphincter retention are associated with a higher survival rate than local excision [10]. The extent of anal resection contains dentine line [11]. The absent anorectal transitional epithelium is replaced by rectum mucosa. Wide perianal soft tissue defect requires bilateral skin flap transfer surgery and anal reconstruction. Patients with PPD combined with other adjacent intestinal malignant tumors may require Miles surgery.
